# Immunity Status of Blood Donors Regarding* Toxoplasma gondii* Infection in a Low-Income District of Abidjan, Côte d'Ivoire, West Africa

**DOI:** 10.1155/2016/6830895

**Published:** 2016-10-03

**Authors:** Liliane Siransy, Sery Romuald Dasse, Serge Pacôme Dou Gonat, Antoinette Legbedji, Koffi N'guessan, Patricia Ama Kouacou, Richard Yeboah, Hervé Menan

**Affiliations:** ^1^Immunology and Allergology Department, UFR Medical Sciences in Abidjan, Felix Houphouet Boigny University, Abidjan, Côte d'Ivoire; ^2^National Blood Transfusion Center, Abidjan, Côte d'Ivoire; ^3^Medical and Technical Parasitology Department, Felix Houphouet Boigny University, Abidjan, Côte d'Ivoire

## Abstract

*Background*. Toxoplasmosis is a widespread cosmopolitan anthropozoonosis, which affects more than a third of the world population. Except the modes of transmission well known,* Toxoplasma gondii* can be transmitted during transplantation or blood transfusion. The aim of this study is to determine the prevalence of IgG and IgM* Toxoplasma gondii* and to estimate the potential risk by blood products.* Methods*. This is a cross-sectional study on the research for* Toxoplasma gondii* antibodies (IgG and IgM) blood donors performed by ELISA.* Results*. An overall seroprevalence of* Toxoplasma gondii* among blood donors recruited was 67.92% (*n* = 72). Among these, 68 have* Toxoplasma gondii* IgG (64.15%), 12* Toxoplasma gondii* IgM (11.32%), and 4 (3.77%) both. The risk varies between 8 for 100000 and 172 for 100000 donations.* Conclusion*. The need to strengthen security measures for people multitransfused, immunocompromised, and pregnant women to reduce the transmission of toxoplasmosis is important.

## 1. Introduction

Toxoplasmosis is a widespread cosmopolitan anthropozoonosis due to a protozoan parasite,* Toxoplasma gondii*, which affects more than a third of the world population [1]. Acquired toxoplasmosis is usually benign in immunocompetent person but can be severe in immunocompromised patients [2, 3]. In practice, in all situations of immunosuppression, existing and planned (chemotherapy, immunosuppressive treatment), HIV status with respect to toxoplasmosis should be established before any prescription that may interfere in the results [4]. In West Africa, particularly in Côte d'Ivoire,* Toxoplasma gondii* infection seroprevalence ranges from 18.2 to 78% [2, 3, 5]. The infection is usually transmitted through the ingestion of food contaminated with oocysts, by eating raw or rare meat and from mother to child [6]. Because of its high frequency and the high proportion of asymptomatic blood donors, we could expect contamination by blood transfusion. However, infections transmitted by blood products are exceptionally documented [7]. Many studies performed among the multitransfused or patients receiving transplants showed a significant difference between the prevalence in cases and that observed in controls [8–13].

In Sub-Saharan Africa in general and in Côte d'Ivoire in particular,* Toxoplasma gondii* infection in blood donors could also represent a risk for transmission in blood recipients but poor information is available. Therefore, the aim of this study is to determine the prevalence of* T. gondii* infection and associated sociodemographic characteristics in a population of healthy blood donors of Abidjan and to estimate the potential risk of transmission of toxoplasmosis by blood products.

## 2. Materials and Methods

We performed a cross-sectional study from October 2014 to December 2014, carried out at the site of Abobo which is a fixed collection site that achieves awareness, recruitment, retention of donors, collection, and storage.

Blood samples were taken from all 106 healthy volunteer and nonremunerated blood donors from the site of Abobo city by successive recruitment.

The tests were then carried out in the laboratory of NBTC. Inclusion criteria for the study were (i) having an age between 18 and 60 years, (ii) being healthy, and (iii) having a weight over 50 Kg.

We used a standardized questionnaire through medical software used routinely at the blood bank named Progesa from MakSystem to explore few characteristics of the blood donors, such sex, age, number of previous donations, ABO and Rh blood group, and occupation.

### 2.1. Analysis

All samples were routinely tested for HIV, HBs Ag, and HCV by ELISA and syphilis by VDRL.

For the detection of anti-*Toxoplasma gondii* antibodies, serum samples were obtained by centrifugation of fresh whole blood taken from the blood donors. The sera were then frozen and stored at −80°C until analysis.

Serological tests for toxoplasmosis were carried out in the laboratory of National Blood Center of Abidjan with commercially available enzyme immunoassay tests* Toxoplasma gondii* IgG ELISA kit and* Toxoplasma gondii* IgM ELISA (Ref: PT-96-Toxo.G lot: 93004/Ref: PT-Toxo.M −96 lot: 93004 of Pishtaz Teb Zaman Diagnostics, Iran) for the detection of immunoglobulin G (IgG) and M (IgM) against* Toxoplasma gondii*. Both tests were performed in the laboratory following the instructions of the manufacturer.

For IgG detection, the test principle is based on indirect ELISA technique in which diluted patient serum samples are allowed to react with coated* Toxoplasma gondii* antigens. The sensibility and specificity were each 100%.

For qualitative calculation, we have distinguished between positive and negative results by the determination of the cut-off index (equal to OD of sample/cut-off value). Based on this formula, results lower than 0.9 were considered as negative and those greater than 1.1 considered as positive results. Those results between 0.9 and 1.1 were considered as suspected results and have been reevaluated.

For quantitative calculation of IgG, a standard curve was constructed point to point by plotting the mean absorbance obtained for each of the four-reference standard against its concentration in IU/mL on linear graph paper, with absorbance on the vertical (*y*) axis and concentration on the horizontal (*x*) axis. The absorbance obtained for each blood donor was projected on the graph and the concentration in UI/mL was obtained by comparison to the standard graph.

For IgM, the test principle is based on antibody capture ELISA technique. The concentration of* Toxoplasma* IgM is directly proportional to the color intensity of the test sample. The sensibility of the test was 100% and the specificity 99%.

To distinguish between positive and negative results the cut-off index was determined: cut-off index = OD of sample/cut-off value.

Based on the above formula, results lower than 0.9 were considered as negative and those greater than 1.1 as positive results. Those results between 0.9 and 1.1 were considered as suspected results and were reevaluated with fresh samples.

Negative results indicate absence of anti-*Toxoplasma* IgM.

Positive results after recheck indicate presence of anti-*Toxoplasma* IgM.

A positive IgG test with a negative IgM test in a donor was interpreted as a chronic infection. A positive IgM test with a positive IgG test in a donor was interpreted as probability of recent infection.

### 2.2. Risk Assessment

To assess the risk contamination in endemic situation of a blood donation by* Toxoplasma gondii*, we applied the mathematical model of the Institute for Public Health Surveillance in France. It was calculated by taking 3% incidence in women of reproductive age living in Abidjan [3]. The risk is equal to the probability of taking a blood donor during parasitemia multiplied by the incidence of infection. The probability of taking such blood donors ranges from one to 21 days of 365 days and the reference incidence is 3% [3, 12].

### 2.3. Statistical Analysis

Statistical analysis of the results was made using Excel 2007, Epi-info 7. For calculation of the sample size, we used Schwartz formula *N* = (*ε* ^2^ *∗p∗q*)/*e* ^2^ with a reference prevalence of 60% [3], a confidence level of 95%, and 0.01 of precision. Descriptive statistics were used for numerical and categorical (percentage) variables. We used the Fisher exact test (if cells values were less than 5) and Pearson test for comparison of the frequencies among groups. For ordinal variables, we used the *χ* ^2^ test for trend.

### 2.4. Ethical Aspects

The national Ethical Committee approved this study. The purpose and procedures of the study were explained to all donors, and a written informed consent was obtained from all of them.

## 3. Results

Demographics characteristics are described in Table 1. 64.15% of donors had* Toxoplasma gondii* antibodies IgG and 32.08% had not yet had contact with* Toxoplasma gondii* (Table 2). The concentration of IgG was calculated for the 68 positive blood donors. The average rate was 96.7 UI/mL with a range of 16.5 IU/mL to 200 IU/mL.

Fourteen (20.59%) subjects had a titer of anti-*Toxoplasma gondii* IgG antibodies between zero and 50 UI/mL, 22 (32.35%) have a titer between 50 and 100 IU/mL (significant titer corresponding to an old immunity or early seroconversion), and 32 (47.06%) an upper title 100 IU/mL (recent seroconversion or a persistent rate).

**Figure 1:**
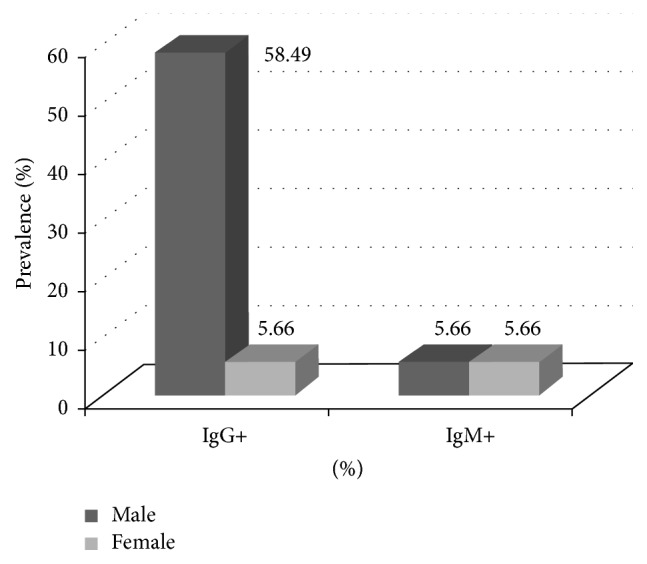
Distribution of* Toxoplasma* antibodies by gender.

**Figure 2:**
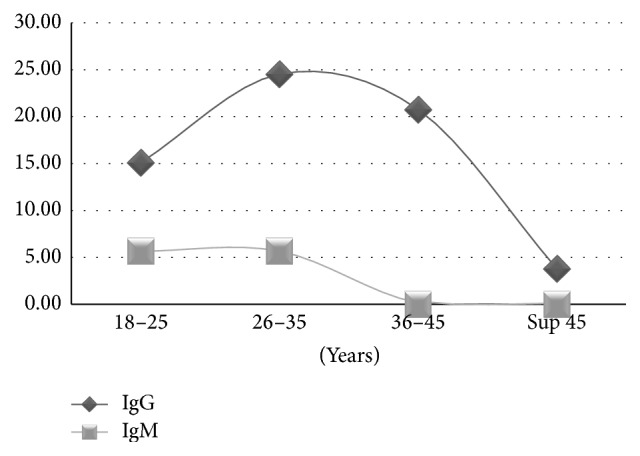
Distribution of* Toxoplasma gondii* antibodies according to the age.

**Figure 3:**
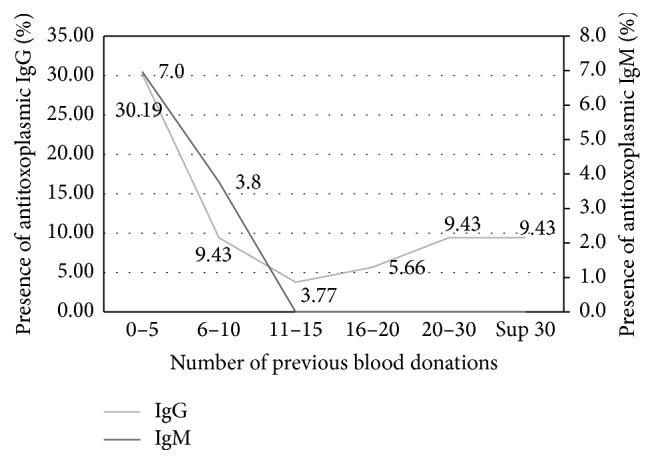
Frequency donor depending on the number of previous blood donations and the presence of IgM and IgG* Toxoplasma gondii* antibodies.

A link was observed between gender and IgM positivity (*p* < 0.05) (Figure 1). According to age, the prevalence of* Toxoplasma gondii* antibodies was lower in older donors (Figure 2) and the highest prevalence of IgG and IgM was found in donors that ranged from zero to five donations (Figure 3).

Regarding blood group, the presence of IgG and IgM* Toxoplasma gondii* antibodies was not linked to the blood donor group. The difference was not statistically significant for IgG and IgM (the exact *p* value based on the Pearson Statistics >0.05). In our sample, 60.38% of RhD positive blood donors had anti-*Toxoplasma gondii* IgG antibodies. The difference is not statistically significant for IgG and IgM (*p* > 0.05) (Table 3).

According to occupation, seroprevalence rate of* Toxoplasma* IgM was higher in students blood donors but differences were not statistically significant (*p* ≥ 0.05) (Table 4).

## 4. Discussion

### 4.1. Demographics of Blood Donors

106 blood donors from the site of Abobo were recruited in our work. This site was chosen because it is located in the most populated city of Abidjan and is considered a city combining the highest rate of low-income district of Abidjan.

Demographic features of the blood donors are shown in Table 1. Male donors were most represented than female donors (sex ratio 6.5). This is explained in large part by the contraindications to the eligibility of blood donation in women with pregnancy, nursing, or menstruating, and the cultural and social constraints in Africa [14].

While 92.45% of donors are regular, 7.5% have no experience of giving blood. Donors who have between one and five previous donations are the most represented with 30.19%. NBTC, through blood safety program, has a policy consisting of retaining the donor for future donations, because a known donor is less at risk than a new donor and an identified donor is a responsible donor [14].

Concerning the occupation, just over one-third or 37.74% are students. Employees are represented by 26.42%, followed by liberal professions 24.53% and 11.32% unemployed. These data are consistent with existing data on blood donors in Côte d'Ivoire* (2014 NBTC Activities report not published)*.

The average age of donors was 31 years with up to 51 years and minimum 20 years. The majority of donors are between 18 and 45 years of which 40% are between 26 and 35 years.

No seropositivity was found for HIV, hepatitis B and C, and syphilis for the blood donors.

### 4.2. Prevalence of Toxoplasmosis in Blood Donors

In this study, the* Toxoplasma gondii* overall seropositivity was 67.92%.

64.15% have* Toxoplasma gondii* IgG antibodies, 11.32% have* Toxoplasma gondii* IgM antibodies, and 3.77% have only IgM without IgG.

The first investigations of toxoplasmosis in Côte d'Ivoire made by Doucet et al. in 1971 found a seroprevalence of 12% [15]. A year later, another survey among women recorded 18.7% seropositivity. Later Dumas, Adoubryn, and Kouakou [2–4] found high prevalence rates ranging from 56.1% to 65.9%. Our figures are in accordance with these rates and testify of the importance of the endemic aspect of the toxoplasmosis in Côte d'Ivoire. This high prevalence can be explained by the differences in the characteristics of the blood donors and environmental aspects.

If we compare the prevalence obtained in donors with neighboring African countries, we find that very little work is done among blood donors concerning toxoplasmosis. In Mali, on 224 blood donors, 49 (21.9%) have* Toxoplasma gondii* antibodies [16].

In west and central Africa, prevalence among the pregnant women ranges from 18.2 to 78% [17], whereas the prevalence among women blood donors in our study is relatively lower because of the weak number of women giving blood donation.

In Libya, in north Africa, the authors worked on women who have had spontaneous abortions and found that 38.5% were seropositive; 36 (66.6%) were positive for IgG antibodies, 12 (22.2%) for IgG and IgM antibodies, and 6 (11.1%) for IgM. Moreover, four of the IgG-positive women had a history of repeated abortion [18].

In France, toxoplasmosis is one of the most prevalent infections with seroprevalence in adults between 20 and 55%, varying according to age, geographic region, and professional category. This prevalence has declined significantly in 30 years with a decline of regular prevalence, currently estimated at 37% [19, 20].

Somewhere else, different works find global toxoplasmosis prevalence going from 7.4% (Mexico), 9.3% (Taiwan), and 19.3% (Iran) to 53.7% (India) [9, 21–23].

#### 4.2.1. Anti-*Toxoplasma gondii* IgG Antibodies

Sixty-eight blood donors on the 106 recruited in our study have* Toxoplasma gondii* IgG antibodies (64.15%). Our rates are lower than Maiga who found 86% of anti-*Toxoplasma gondii* IgG antibodies among HIV negative blood donors and 100% in HIV seropositive blood donors [16].

#### 4.2.2. Anti-*Toxoplasma gondii* IgM Antibodies

The presence of anti-*Toxoplasma* IgM reflects the risk of transmission by blood transfusion. In this current study, 11.32% of donors had IgM antibodies.

In a study conducted by Sarkari in Iran, 81 donors in 1480 (5.47%) had anti-*Toxoplasma gondii* IgM antibodies. All these donors were tested for the DNA of the parasite that was found in two blood donors to be 1.9%. Other authors have conducted a real-time PCR and all samples were negative [23].

Donors having anti-*Toxoplasma gondii* IgM antibodies present a significant risk of acute infection and thus can transmit the disease but its presence does not necessarily indicate an acute infection [23].

### 4.3. Study of the Variables Associated with* Toxoplasma gondii* Seroprevalence

#### 4.3.1. Sex

The prevalence of anti-*Toxoplasma gondii* IgG antibodies was highest among male donors. Of the 92 male blood donors, 58.49% had* Toxoplasma gondii* IgG antibodies and 5.66%* Toxoplasma gondii* IgM antibodies.

As for female donors, 5.66% prevalence of both IgG and IgM was found. This prevalence was much lower than that found in pregnant women in Côte d'Ivoire [2–4], Burkina Faso (31%) [24], Gabon (56%) [25], and Morocco (50.6%) [26]. This difference was probably related to the selection of blood donor before donation, to assess their eligibility to donate before retaining them as donor.

The sex was not associated with seropositivity for IgG. However, it is difficult to confirm any relationship between gender and* Toxoplasma gondii* antibodies prevalence since more than 80% of donors in our study are male.

#### 4.3.2. Age

In a global way, in this current study, the more our donors are old, the less the prevalence is high. These data contradict those of Sarkari et al. in Iran and El Mansouri et al. in Morocco [9, 26] who studied the variation of toxoplasmosis' seroprevalence in women according to age. The percentage of positivity of* Toxoplasma gondii* IgG was 32.4% among women under 20 years of age while it was 52% in women between 20 and 39 years. In women over 40 years, the rate was 63.8%. Adoubryn et al. [3] and Sarkari et al. [9] made the same assessment: the rates of seropositivity increase with the age.

However, Chiang et al. [22] and Elhence et al. [23] find no correlation between age and the presence of* Toxoplasma gondii* antibodies.

#### 4.3.3. Number of Previous Donations

A peak for both IgG and IgM was observed in donors having between zero and five blood donations. This is in perfect harmony with the seroprevalence of markers usually found among blood new donors as HIV antibodies, HBsAg (hepatitis B), and anti-HCV (hepatitis C) antibodies. A new donor is more at risk than a known and identified donor [14].

#### 4.3.4. Toxoplasmosis, Blood Groups, and Profession

We observed that IgM anti-*Toxoplasma gondii* seropositivity is high in students and unemployed donors maybe due to precarious living conditions, poverty, and undernourishment.

Havlícek et al. [27] in a double-blind study shows that there is a longer reaction time in people with latent toxoplasmosis. The existence of a positive correlation between length of infection and mean reaction time suggested that slow and cumulative effects of latent toxoplasmosis are responsible for the decrease of psychomotor performance of infected subjects.

One study conducted in 3900 military drivers shows that* Toxoplasma gondii* infection increased the risk of traffic accidents in military drivers. In RhD negative subjects, the probability of traffic accidents increased with titer of anti-*Toxoplasma* antibodies [28].

In our study, no correlation was found between* Toxoplasma* seropositivity and ABO or RhD blood group (*p* > 0.05).

### 4.4. Residual Risk of Transfusion Transmission of Toxoplasmosis

The existence of an asymptomatic blood passing certain viruses, bacteria, or parasites leads to a risk of transmission of these agents during a blood transfusion especially patients undergoing multiple transfusion.

Few studies are available on contaminated blood* Toxoplasma gondii* in humans. In mice,* Toxoplasma gondii* was injected and developed toxoplasmosis. These mice were then blood donors for other mice that have been contaminated [29].

In Sarkari et al. study in Iran, PCR detected active parasitemia in two (1.9%) of the IgM-positive subjects. The presence of parasitemia revealed by PCR in IgM-positive healthy blood donors ensures the likelihood of transmission of* Toxoplasma* through blood transfusion.

Presence of organism in blood during the course of infection ensures its transmission through transfusion [9]. Moreover, the ability of organism to survive in the stored blood is another factor which increases the chance of transmission through transfusion. It has been found that tachyzoites of* Toxoplasma* can survive in stored blood for several weeks [9].

If this risk is currently well controlled for some infectious agents (HIV, HCV, and HBV), it is not sufficiently documented and quantified for* Toxoplasma gondii*, especially since toxoplasmosis may be increased upon the occurrence of an outbreak. Larger epidemics are possible in case of water contamination, for example, and it is then of 2 to 5 times higher than the incidence of endemic situation.

Symptomatic donor is excluded from donating systematically donation at the clinic predonation selection. This risk depends on the exclusion criteria of prospective donors and the likelihood that the donor is infectious and asymptomatic the day of donation.

To study the risk of blood transfusion, Ebrahim Zadeh and Stuart et al. [13, 30] investigated the seroprevalence in multitransfused hemodialysis patients. He observed a high prevalence of* Toxoplasma* antibodies in patients exposed to transfusion against lower in the control group and found that hemodialysis patients should be regularly monitored to avoid the risk of acute toxoplasmosis.

In addition, the French Institute of Health Surveillance proposed in 2005 a quantitative estimate of the risk of contamination of a blood donation by infectious agents to assess the risk of contamination of donated blood by the pathogen of toxoplasmosis [12].

In our study, we applied the mathematical model of the Institute for Public Health Surveillance in France. It was calculated by taking 3% incidence in women of reproductive age living in Yopougon [3] and thus varies between 8 per 100 000 and 172 per 100 000 for donations (Table 5). In France, in 2003, it ranged between 1.37 per 100,000 and 28.8 per 100,000 donations which is 7 times lower.

However, it does not estimate the real risk of transmission to the recipient, as it only takes into account neither the transmission efficiency, nor the effectiveness of process for preparing blood components, nor the recipient immunity. For example, the ability of tachyzoites of* Toxoplasma* to survive in stored blood for several weeks is a factor increasing the risk of transmission by blood transfusion [12]. Using leucocytes reduced blood may be an option to reduce the risk in immunocompromised patients. All suspicious blood products were discarded because units are not routinely leukoreduced in our blood bank and the blood donors were addressed to the “Centre de suivi des donneurs” and followed by a medical doctor.

## 5. Conclusion

Our manuscript highlights seroprevalence of anti-IgG and IgM* Toxoplasma gondii* in blood donors and the potential risk of transmission of toxoplasmosis by blood products. Symptomatic donors are systematically excluded from donating at the clinic predonation selection but, in the majority, toxoplasmosis is clinically unapparent.

In our study, the risk of transmitting blood components contaminated with* Toxoplasma gondii* can reach 172 for 100000 donations. These data indicate a high level of endemicity in our blood donors, particularly to those having less than five blood donations.

Effective strategies are required to prevent transfusion-transmitted toxoplasmosis. Because of lack of funding in our country, the total number enrolled in our study is small and recommend us to be careful in interpretation.

Toxoplasmosis test does not need to be obligatory; we rather recommend education programmes for blood donors and systematic leucocytes reduced blood for people multitransfused, immunocompromised, and pregnant women* T. gondii* antibody-negative blood components for transfusion to avoid transmission by blood products. We think that more prospective studies with PCR need to be conducted across our country for a better comprehension and organization of programmes to fight against transmission of* Toxoplasma gondii* by blood products.

## Figures and Tables

**Table 1:** Distribution of donors according to demographic characteristics.

	Number	%
*Gender*		
Male	92	86.79
Female	14	13.21
*Age*		
18–25 years	34	32.08
26–35 years	40	37.74
36–45 years	26	24.53
>45 years	6	5.66
*Number of previous donations*		
0	8	7.55
1–5	32	30.19
6–10	18	16.98
11–15	12	11.32
16–20	10	9.43
21–30	14	13.21
>30	12	11.32
*Occupation*		
Liberal	26	24.53
Employed	28	26.42
Unemployed	12	11.32
Students	40	37.74
*Blood group ABO Rh*		
O	62	58.49
B	30	28.30
A	12	11.32
AB	2	1.89
Rh D positive	94	88.68
Rh D negative	12	11.32
*Anti-Toxoplasma gondii antibodies IgG *		
IgG positive	68	64.15
IgG negative	38	35.85
*Anti-Toxoplasma gondii antibodies IgM*		
IgM positive	12	11.32
IgM negative	94	88.68

**Table 2:** Distribution of donors by the immune profile *Toxoplasma* IgG and IgM.

	Presence of *Toxoplasma gondii* IgG antibodies (IgG+)		Absence of *Toxoplasma gondii* IgG antibodies (IgG−)		Total	
	Number	%	Number	%	Number	%
Presence of *Toxoplasma gondii* IgM antibodies						
*(IgM+)*	8	*7.55*	4	*3.77*	12	*11.32*
Absence of *Toxoplasma gondii *IgM antibodies						
*(IgM−)*	60	56.60	34	32.08	94	88.68

*Total*	*68*	*64.15*	*38*	*35.85*	*106*	*100*

**Table 3:** Distribution of donors according to the ABO-Rh and the presence of IgM and IgG *Toxoplasma gondii* antibodies.

ABO blood group	Number of blood donors	(%)	Presence of antitoxoplasmic IgG		Presence of antitoxoplasmic IgM	
			Number	%	Number	%
O	62	58.49	40	37.74	6	5.66
B	30	28.30	22	20.75	4	3.77
A	12	11.32	6	5.66	2	1.89
AB	2	1.89	0	0.00	0	0.00

*Total*	*106*	*100*	*68*	*64.15*	*12*	*11.32*

RhD−	12	11.32	4	3.77	0	0.00
RhD+	94	88.68	64	60.38	12	11.32

*Total*	*106*	*100*	*68*	*64.15*	*12*	*11.32*

**Table 4:** Distribution of donors depending on the profession and the presence of IgM and IgG *Toxoplasma gondii* antibodies.

Occupation	Number of blood donors	%	Presence of IgG *Toxoplasma * *gondii* antibodies		Presence of IgM *Toxoplasma * *gondii* antibodies	
			Number	%	Number	%
Liberal profession	26	24.53	16	23.53	0	0
Employed	28	26.42	26	38.24	2	16.67
Unemployed	12	11.32	4	5.88	4	33.33
Student	40	37.74	22	32.35	6	50

Total	*106*	100.00	*68*	100.00	*12*	100

**Table 5:** The quantitative estimation of risk of contamination of a blood donation by *Toxoplasma gondii* in endemic situation.

Items	Low hypothesis	High hypothesis
Number of days	365	365
Duration of parasitemia (days)	1	21
Probability to take a donor in parasitemia stage	1/365	21/365
Incidence of infection%	3	3
*Risk of infected donation for 100000 donations*	*8.22*	*172.60*
